# The effects of demographic stochasticity and parameter uncertainty on predicting the establishment of introduced species

**DOI:** 10.1002/ece3.2495

**Published:** 2016-10-27

**Authors:** Gian Marco Palamara, Francesco Carrara, Matthew J. Smith, Owen L. Petchey

**Affiliations:** ^1^ Department of Evolutionary Biology and Environmental StudiesUniversity of ZurichZürichSwitzerland; ^2^ Computational Science LaboratoryMicrosoft ResearchCambridgeUK; ^3^ Ralph, M. Parson Laboratory, Department of Civil and Environmental EngineeringMassachusetts Institute of TechnologyCambridgeMAUSA; ^4^ Department of Civil, Environmental and Geomatic EngineeringETHZürichSwitzerland

**Keywords:** Bayesian inference, birth and death process, demographic stochasticity, establishment probability, invasion dynamics, Lotka–Volterra equations

## Abstract

Invasive species are a serious threat to biodiversity worldwide and predicting whether an introduced species will first establish and then become invasive can be useful to preserve ecosystem services. Establishment is influenced by multiple factors, such as the interactions between the introduced individuals and the resident community, and demographic and environmental stochasticity. Field observations are often incomplete or biased. This, together with an imperfect knowledge of the ecological traits of the introduced species, makes the prediction of establishment challenging. Methods that consider the combined effects of these factors on our ability to predict the establishment of an introduced species are currently lacking. We develop an inference framework to assess the combined effects of demographic stochasticity and parameter uncertainty on our ability to predict the probability of establishment following the introduction of a small number of individuals. We find that even moderate levels of demographic stochasticity influence both the probability of establishment, and, crucially, our ability to correctly predict that probability. We also find that estimation of the demographic parameters of an introduced species is fundamental to obtain precise estimates of the interaction parameters. For typical values of demographic stochasticity, the drop in our ability to predict an establishment can be 30% when having priors on the demographic parameters compared to having their accurate values. The results from our study illustrate how demographic stochasticity may bias the prediction of the probability of establishment. Our method can be applied to estimate probability of establishment of introduced species in field scenarios, where time series data and prior information on the demographic traits of the introduced species are available.

## 1 Introduction

Introduced species that successfully establish and then become invasive are one of the major threats to biodiversity (Cardinale et al., 2006; Chapin et al., 2000; Pereira et al., 2010). Understanding the dynamics of invasions has become a matter of major interest due to an increase in the number of cases of invasive species and the economic costs and environmental damage that they cause (Cacho, Spring, Pheloung, & Hester, 2006; Simberloff et al., 2013; Vitousek, Antonio, Loope, & Westbrooks, 1996). The establishment success of introduced species has been intensively studied for a variety of organisms such as microbes (Litchman, 2010), plants (Corbin & D'Antonio, 2004; Keane & Crawley, 2002; Vilà et al., 2011), insects (Kenis et al., 2008), fishes (Kolar & Lodge, 2002), and birds (Blackburn, Cassey, & Lockwood, 2009; Cassey, Blackburn, Sol, Duncan, & Lockwood, 2004). Previous studies have shown that the success rate of establishment of an introduced species depends on several factors (Tilman, 2004; Williamson & Fitter, 1996), including the traits of the introduced species (Van Kleunen, Dawson, Schlaepfer, Jeschke, & Fischer, 2010; Williamson & Fitter, 1996) and its interactions with the resident species (Bright, 1998; Jeschke & Strayer, 2006), the founding population size (Duncan, Blackburn, Rossinelli, & Bacher, 2014), environmental conditions (Beisner, Hovius, Hayward, Kolasa, & Romanuk, 2006), the ability of the introduced species to adapt to the new habitats (Blackburn et al., 2009; Keane & Crawley, 2002; Sax et al., 2007), the complexity of the food web of the resident community (Lurgi, Galiana, López, Joppa, & Montoya, 2014; Romanuk et al., 2009), and the spatial structure of the landscape (With, 2002). Given the variety of interdependent mechanisms, understanding why some introduced species establish while others fail remains a central challenge in invasion ecology (Sax et al., 2007).

Invasions dynamics, like many other ecological processes, are typically regarded as random processes. Accordingly, an estimate of the probability of establishment of the introduced species in the resident community is needed in order to make predictions. In the early stages of an introduction, the population size of the introduced species is often small and demographic stochasticity plays an important role in determining whether an introduced species establishes (Drake et al., 2006). In the past, theoretical studies have often treated species invasion as a deterministic process (e.g., Galiana, Lurgi, Montoya, & López, 2014; Lurgi et al., 2014; Romanuk et al., 2009; Yodzis & Innes, 1992). However, deterministic models, by ignoring the intrinsic stochasticity of biological processes, can lead to different predictions from models that explicitly include such stochasticity (Carrara, Giometto, Seymour, Rinaldo, & Altermatt, 2015; Ebenman, Law, & Borrvall, 2004; McKane & Newman, 2004 McKane & Newman, 2005). For example, fluctuations in the speed of an advancing population wave can be estimated by knowing demographic stochasticity, which is dominant at the leading edge of the front (Giometto, Rinaldo, Carrara, & Altermatt, 2014). Furthermore, demographic stochasticity can slow down biological invasions (Snyder, 2003) resulting in smaller invasion speeds than the one predicted by simpler deterministic models (Elliott & Cornell, 2013).

Together with demographic stochasticity, biotic interactions affect the probability of establishment (Bulleri, Bruno, & Benedetti‐Cecchi, 2008). An introduced species can interact in several ways with the local pool of resident species (Shea & Chesson, 2002). The invader can be a predator, a competitor or a facilitator for some of the species in the resident community. Competitive interactions are particularly relevant in determining the establishment success rate of an introduced species (Case, 1990; Corbin & D'Antonio, 2004; Tilman, 2004). Using deterministic models of competition, it has been shown that the probability of invasion success decreases with the size of the resident community, and the average strength of competition between the residents and the introduced species (Case, 1990).

Understanding the combined effects of demographic stochasticity and species traits, such as typical demographic rates and competitive interaction strengths, is fundamental to predicting the establishment of an introduced species (Lande, Enger, & Sæther, 2009). However, characterizing the properties and relative strengths of these processes requires information on the ecological traits of the introduced individuals in their new habitat. To infer the probability of species establishment in the field, it is useful to combine empirical field observations with laboratory experiments and mathematical modeling using simple model systems (Altermatt et al., 2015). Early approaches involved experimental microcosms of two competing species at the dynamical equilibrium, and the competition strengths were inferred by assuming a simple Lotka–Volterra competition models (Case, 1990; Leslie, 1957). Later methods relaxed the equilibrium assumption and used the transient population dynamics to infer competition parameters, either with deterministic (Pascual & Kareiva, 1996) or stochastic models in single‐ and multispecies scenarios (Boys, Wilkinson, & Kirkwood, 2007; Carrara et al., 2015; Gilioli, Pasquali, & Ruggeri, 2008; Palamara et al., 2014).

Inferred information about ecological processes will always include errors. This is likely to be present when inferring information about introduced species, for which there will typically be limited information, particular about how they interact with resident species. Recently, Palamara et al., 2014 revealed how constraints in data availability and assumptions about the underpinning ecological dynamics combine to influence the precision and accuracy with which population parameters can be inferred for a single species population undergoing logistic growth. If such a species establishes then we would expect that uncertainty about the population parameters would propagate into uncertainty about the probability of a successful invasion. This leads to a more general question about the ability to predict species invasions: if we infer properties about the ecological processes underpinning establishment with uncertainty and we treat establishment as a probabilistic process then how does the uncertainty in the inferred parameters influence our ability to accurately predict the probability of establishment? Advances in this area are needed to improve our understanding of the extent to which we can accurately forecast the dynamics of different types of ecological systems (Petchey et al., 2015).

In this article, we investigate how interaction between uncertainty about population parameters and demographic stochasticity affect our ability to accurately predict the probability of the successful establishment of an introduced species in a community of resident species, using mathematical models to produce theoretical dynamics of the resident and introduced species. We focus on demographic stochasticity as a source of randomness in species population dynamics because it is common to most species introductions. First, we derive novel analytical approximations to estimate the interaction strength between the resident species and the introduced one, during the early stages of the introduction. We assess the establishment probability of an introduced species as a function of the inferred competition parameter and the demographic parameters of the introduced species. We then use Bayesian parameter inference techniques to infer our predictive ability in the estimation of the establishment of an introduced species. Furthermore, we test the dependence of the predictive ability on uncertainty of the introduced species' demographic parameters.

## 2 Methods

We model the establishment dynamics with a stochastic version of the generalized Lotka–Volterra equations for interspecific competition, in the form of a continuous time birth and death processes (BDP), which enables us to explicitly take into account demographic stochasticity (Black & McKane, 2012; Nåsell, 2001). To reduce model complexity, in the limit of many resident species, we study the establishment dynamics by a linearization of the interaction term. We work under the hypothesis that the abundance of the resident community is very large compared to the introduced species abundance. This enables us to analyze the population equations as a singular perturbation theory problem, where the perturbation is the small effect of the small introduced species population. We use this to derive a diffusion approximation (Ross, Pagendam, & Pollett, 2009) for the probability distribution of the introduced species' population size as a function of time that includes the effect of resident‐introduced competition. This probability distribution is used to derive a likelihood function for a set of observations of the introduced population size given a particular set of demographic parameters. We use this likelihood function to infer the probability distributions of the introduced species demographic parameters (birth and death rates, carrying capacity, and competition coefficient) given measurements of the introduced population size over time.

By the predictability of introduction, we mean the degree to which a prediction of a given outcome of the introduction can be expected to be correct. In our case, the only outcomes we consider are whether the introduced population has become extinct or not, where *p*(extinction by time *t*) = 1 − *p*(establishment by time *t*), and by establishment, we simply mean that the introduced species has not yet gone extinct. In reality, all species will eventually go extinct after sufficient time has elapsed because of demographic fluctuations; thus, eventual extinction is perfectly predictable (strictly speaking, the only stable state is the extinction state, but here we are only looking at meta‐stable states). The corollary is that an introduced species can only be said to establish if its population has not gone extinct deterministically after a given time. In our study, we use the probability of species extinction and establishment after a certain time period as a measure of the predictability of those events. For example, if the probability that the species has gone extinct by 10 days is 0.5 then the predictability of extinction is 0.5 (if someone had placed a bet on extinction then they would win 50% of the time). By predictive ability, we mean our expectation about our ability to predict the correct possible outcome. While predictive ability may exceed or be lower than predictability by chance, on average, predictive ability is fundamentally constrained by the predictability and is likely to be lower because the model represents the real system imperfectly. Using simulated data, we investigated how the predictability and the predictive ability of the introduction dynamics are affected by both demographic noise and the interaction between the resident community and the introduced species.

### 2.1 Model

Given a community of *S* species, under the assumption that every species in the community can interact with any other, the linearized set of differential equations

(1) $$\frac{dn_{i}}{dt}=r_{i}n_{i}\left(1-\frac{n_{i}+\sum_{j\neq i}^{S}\alpha_{ij}n_{j}}{K_{i}}\right)$$

is the classic Lotka–Volterra community model (Ebenman, Law, & Borrvall 2004),where, $n_{i}\left(t\right)$ is the population density of species *i*,* r*_*i*_ is its the per capita growth, *K*_*i*_ is its carrying capacity, and α_*ij*_ is the per capita effect of species *j* on species *i* (α_*ii*_ = 1). For an introduced species *I*, Equation 1 can be generalized by a stochastic BDP represented by the following birth and death rates

(2) $$\begin{array}{cc} B(n_{I}) & =\lambda_{I}n_{I}\left[1-\left(\frac{n_{I}+\sum_{j\neq I}^{S}\alpha_{Ij}n_{j}}{N_{I}}\right)\right]\\ D(n_{I}) & =\mu_{I}n_{I} \end{array}$$

where λ_*I*_ and μ_*I*_ are the per capita intrinsic birth and death rates of the introduced species (*r*_*I*_ = λ_I_ − μ_*I*_) and *N*_*I*_ is the population size at which there is zero probability of births for the introduced species (Nåsell, 2001). In process 2, the carrying capacity of the introduced species *K*_*I*_ is explicitly separated from its maximum population size *N*_*I*_, which in turn is a physical limit related to the size of the habitat. *N*_*I*_ and *K*_*I*_ are related by the intrinsic birth and death rates, which define the maximum size of the demographic fluctuations at equilibrium (*K*_*I*_ = *r*_*I*_ *N*_*I*_/λ_*I*_). Demographic stochasticity becomes relevant for population dynamics when population size is small compared to the maximum population size ($n_{I}O\left(\sqrt{N_{I}}\right)$) as is the case during the first stages of an introduction.

Considering the population density of the introduced species *n*_*I*_ and omitting terms of order *O*(1/*S*) or smaller, we rewrite Equations 1 for the introduced species, *I*, as

(3) $$\frac{dn_{I}}{dt}\approx \frac{r_{I}n_{I}}{K_{I}}\left(K_{I}-n_{I}+\alpha_{IR}n_{R}+\sum_{j\neq I}^{S}\left(S-1\right)\text{cov}\left(n_{j},\alpha_{Ij}\right)\right)$$

where *r*_*I*_ and *K*_*I*_ are the growth rate and carrying capacity of the introduced species, $\alpha_{IR}=\sum_{j\neq I}\alpha_{I}j$ is the overall effect of the species in the resident community on the introduced species, *n*_*R*_ is the average species density of the resident community species, and $\text{cov}(n_{j},\alpha_{Ij})$ is the covariance measured over the densities of the resident species and the introduced species' interaction strengths. In the mean field approximation of a community with many species, the covariance term is negligible and so we omit it from our analysis. In order to decouple the system of Equation 3, we assume that the species in the resident community remain at their average density, that is, at carrying capacity, *K*_*R*_. This assumption holds by considering the earlier stages of an introduction, where it can be shown that the resident species remain close to the average density, *n*_*R*_ > 0.99 *K*_*R*_ (see also Section 2.2). We can now rewrite process 2 as a single species BDP for the introduced species with a modified birth rate given by

(4) $$\begin{array}{cc} B(n_{I}) & ≃{\overset{¯}{\lambda }}_{I}n_{I}\left(1-\frac{n_{I}}{N_{I}}\right)\\ D(n_{I}) & =\mu_{I}n_{I} \end{array}$$

where

(5) $$\bar{\lambda_{I}}=\lambda_{I}\left(1-\frac{\alpha_{IR}K_{R}}{N_{I}}\right)$$

is the modified intrinsic birth rate of the introduced species, which takes into account the effect of the interaction between the introduced and the resident species. The approximated process 4 is a convenient version of the stochastic logistic equation (Nåsell, 2001; Ross, Taimre, & Pollett, 2006) with a modified growth rate (${\overset{¯}{r}}_{I}={\overset{¯}{\lambda }}_{I}-\mu_{I}$) and carrying capacity (${\overset{¯}{K}}_{I}={\overset{¯}{r}}_{I}N_{I}/{\overset{¯}{\lambda }}_{I}$) (see also section 6 for a more general formulation of process 4).

The Lotka–Volterra deterministic model has four possible solutions, which are obtained by varying the interaction parameters in the range$\left[\alpha_{IR},\alpha_{RI}\right]\in \left[0,2\right]\times \left[0,2\right]$: stable coexistence ($\left[\alpha_{IR},\alpha_{RI}\right]\in \left[0,1\right]\times \left[0,1\right]$),unstable coexistence ($\left[\alpha_{IR},\alpha_{RI}\right]\in \left[1,2\right]\times \left[1,2\right]$), resident goes extinct ($\left[\alpha_{IR},\alpha_{RI}\right]\in \left[0,1\right]\times \left[1,2\right]$), introduced species goes extinct ($\left[\alpha_{IR},\alpha_{RI}\right]\in \left[1,2\right]\times \left[0,1\right]$). Any other choice of parameter values can be converted to those values by an appropriate renormalization of the competition coefficients and the carrying capacities. We chose $\alpha_{RI}=0.5$ so that the possible dynamics are reduced to one of the two possible outcomes for the deterministic solution: (1) the introduced species establishes ($\alpha_{IR}\in \left[0,1\right]$), or (2) the introduced species does not establish ($\alpha_{IR}\in \left[1,2\right]$). At $\alpha_{IR}=1$ (hereafter, we will refer to the interaction parameter simply as α), there is a bifurcation in the deterministic solution (Gardiner, 2009). On average, an introduced species that is predicted to establish by the deterministic solution tends to increase in abundance over time after initial introduction, whereas a species that is predicted to not establish by the deterministic solution on average tends to have a negative population growth rate to extinction.

### 2.2 Stochastic simulations

We simulated the stochastic process (2) using the Gillespie algorithm (Gillespie, 1977), along a gradient of demographic stochasticity, where a low level of demographic stochasticity results in relatively small population fluctuations about the expected trajectory and high demographic stochasticity results in relatively high fluctuations. We varied demographic stochasticity independently of the mean dynamics of the population in process 4 by recognizing that the difference of the birth and death rates (i.e., birth rate–death rate) of the population defines the mean trajectory of the population, whereas their sum (i.e., birth rate + death rate) defines the extent of demographic stochasticity (Nisbet & Gurney, 1982). We introduce the parameter $\delta =\frac{\lambda_{I}+\mu_{I}}{2}=\frac{\lambda_{R}+\mu_{R}}{2}$ to control demographic stochasticity (Gardiner, 2009; Ovaskainen & Meerson, 2010). Therefore, the size of demographic fluctuations, given by the sum of birth and death rates (Ovaskainen & Meerson, 2010), is proportional to the parameter δ (Gardiner, 2009). We simulated the process 4 with different strengths of demographic stochasticity, by varying δ∈[0.5,5], where $\lambda_{I}=\lambda_{R}=\delta +0.5$ days^−1^, $\mu_{I}=\mu_{R}=\delta -0.5{\text{days}}^{-1}$ and $N_{I}=N_{R}=10000\left(\delta +0.5\right)\;individuals$.In this way, the mean growth rate and the carrying capacity of the resident and the introduced species were fixed (*r*_*I*_ = *r*_*R*_ = 1 days^−1^ and *K*_*I*_ = *K*_*R*_ = 10,000 individuals). This ensured that, for any interaction coefficient, the resident species $n_{R}\left(t\right)>0.99K_{R}$, for *t *< 5 days, which corresponds on average to >7 generations for the invader. See table 1 for a complete list of the simulation parameters and their values

**Table 1:** List of simulation parameters

Parameter	Description	Values used in simulations
δ	Coefficient of demographic stochasticity	0.5–5 days^−1^
*N*_*I*_	Maximum population size of invader species	10,000 (δ + 0.5) individuals
*N*_*R*_	Maximum population size of resident species	10,000 (δ + 0.5) individuals
λ_*I*_	Intrinsic birth rate of invader species	δ + 0.5 days^−1^
λ_*R*_	Intrinsic birth rate of resident species	δ + 0.5 days^−1^
μ_*I*_	Intrinsic death rate of invader species	δ − 0.5 days^−1^
μ_*R*_	Intrinsic death rate of resident species	δ − 0.5 days^−1^
*r*_*I*_ = λ_*I*_ − μ_*I*_	Growth rate of the invader	1 days^−1^
*r*_*R*_ = λ_*R*_ − μ_*R*_	Growth rate of the resident	1 days^−1^
$K_{I}=\frac{r_{I}N_{I}}{\lambda_{I}}$	Carrying capacity of the invader	10,000 individuals
$K_{R}=\frac{r_{R}N_{R}}{\lambda_{R}}$	Carrying capacity of the resident	10,000 individuals
α_*RI*_	Per capita effect of invader on resident	0.5
α_*IR*_	Per capita effect of resident on invader	0.5 − 1.5
n_0*I*_ = n_0_	Initial population size for the invader	40 individuals
n_0*R*_	Initial population size for the resident	10,000 individuals
σ	Prior variance	0.01−1

**Figure 1:**
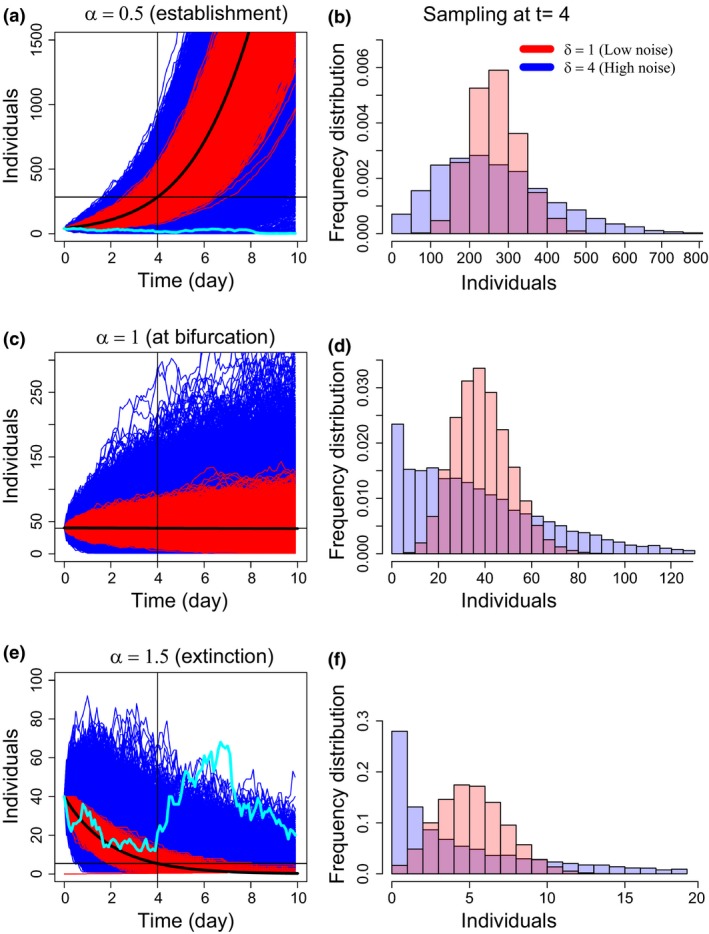
Time series of number individuals from 10,000 replicates of the stochastic process 2 for three different parametrization: (a) α = 0.5, which causes the introduced species to successfully establish in the deterministic model; (c) α = 1, for which successful establishment in the deterministic model is dependent on initial conditions; and (e) α = 1.5, for which the introduced species always declines to extinction in the deterministic model. The thick black lines in (a, c, e) show the predicted deterministic dynamics. Red corresponds to simulations with low demographic noise (δ = 1) and blue to high demographic noise (δ = 4). In cyan, we highlighted two trajectories of a single population whose size at t = 10 is opposite from the deterministic expectations. (b, d, f) Distributions of population sizes of the introduced species for the three cases of the panels a, c, e, at *t* = 4 days. Initial conditions are *K*_*R*_ = 10,000 for the resident community (carrying capacity) and *n*_*I*_ = 40 individuals for the invader

See Figure 1 for processes at different values of demographic noise, representing a realistic range of possible population fluctuations. The Gillespie algorithm produces a continuous time series of population sizes, where demographic stochasticity is reproduced through the stochastic simulation of individual birth and death events. For simplicity, we keep fixed the size of the resident community. As initial conditions, we always set the resident community at the average density *K*_*R*_ = 10,000. The choice of initial population size of the introduced species (*n*_0_ = 40) is large enough to be consistent with our approximations, that is, of the order of $\sqrt{N_{I}}$ and values of *n*_0_ > 40 individuals may not affect our conclusions (for *n*_0_ = 40 individuals and for the highest value of δ (5 days^−1^) establishment probability has already saturate [fig. 1a of Duncan et al., 2014]). Please refer to Duncan et al., 2014 and to Kramer & Drake, 2014 for a systematic investigation of the effect of varying the initial population size of the invader in this regime.

### 2.3 Likelihood function

To conduct parameter inference, we need a mathematical function defining the probability of a set of parameters given the data, that is, the likelihood function. The likelihood function takes as arguments the data and $\theta =(\log\left(r_{I}\right),\log\left(K_{I}\right),\log\left(N_{I}\right),\log\left(K_{R}\right),\alpha )$, that is, the parameters of process 4. Time series data for the population of the introduced species (hereafter labeled as $n_{I}\equiv n$) are given by $y=(n_{0},t_{0};n_{1},t_{1};\ldots ;n_{d},t_{d})$,where *d* is the number of observations (the sampling effort), and *n*_*i*_ is the number of individuals counted at time *t*_*i*_. Ross et al., (2009) derived a diffusion approximation for the BDP 4 (assuming the maximum population size *N* is sufficiently large), which gives the probability of observing a particular number of individuals, *x*, as a Gaussian distribution with time dependent mean and variance

(6) $$P_{g}\left(X=x;t\right)=\frac{1}{\sqrt{2\pi \sigma {\left(t\right)}^{2}}}\exp\left(-\frac{x-n(t)}{2\sigma {\left(t\right)}^{2}}\right)$$

where the mean of this distribution, *n*(*t*), is given by the solution of the logistic equation

(7) $$n\left(t,n_{0}\right)=\frac{Kn_{0}e^{rt}}{K+n_{0}\left(e^{rt}-1\right)}$$

and the variance is given by

(8) $$\sigma \left(t,n_{0}\right)=NM_{t}^{2}\int_{0}^{t}H\left(n\left(s,n_{0}\right)/N\right)M_{s}^{-2}ds$$

where $M_{s}=\exp\int_{0}^{s}C_{s}ds$, $C_{s}=F^{\prime }\left(n\left(s\right)/N\right)$ (Ross et al., 2006, 2009). The two functions, *F* and *H*, are given by F(n)=B(n)−D(n) and H(n)=B(n)+D(n), where *B* and *D* are the birth and death rates of process 4.

The likelihood function is thus given by

(9) $$L\left(y|\overset{¯}{y},\theta \right)=\sum_{i=1}^{d}\log\left[P_{g}\left(X={\overset{¯}{n}}_{i};t_{i},\theta \right)\right]+\log\left[P_{f}\left(X=n_{i},{\overset{¯}{n}}_{i}\right)\right]$$

where *P*_*g*_ is the normal distribution with mean and variance parametrized with **θ**, and *P*_*f*_ is a Poisson distribution describing the probability of observing a population of $\overset{¯}{n}$ individuals when the actual size of the population is *n*, and the fraction of habitat searched is *f* (*f* < 1). The first term of the likelihood 9 describes the demographic process 4 and the second term describes the sampling error associated to any experiment or field survey.

We use a Bayesian framework to obtain the estimates of the parameters of the model, and we investigate the effect of having a prior knowledge of the parameters on their estimate. Bayes theorem (Gilks, Richardson, & Spiegelhalter, 1996) enables the calculation of the joint probability distribution of the parameters of the model given the data and translated into our parameter estimation problem it is

(10) $$P\left(\theta ,\overset{¯}{y}|y\right)=\frac{L\left(y|\overset{¯}{y},\theta \right)P_{\theta }P_{\overset{¯}{y}}}{\int L\left(y|\overset{¯}{y},\theta \right)P_{\theta }P_{\overset{¯}{y}}d\theta d\overset{¯}{y}}$$

where *P*_**θ**_ and $P_{\overset{¯}{y}}$ are the prior probability distributions for the parameters of process 4 and for the observed population sizes, respectively. The term on the denominator (the marginal likelihood) is irrelevant in this study, and we set it equal to 1 throughout. We always put uniform uninformative priors for $P_{\overset{¯}{y}}$ and we test different prior scenarios for *P*_**θ**_ (see section 5). We use Markov chain Monte Carlo sampling with the Metropolis–Hastings algorithm (Chib & Greenberg, 1995), implemented using the software Filzbach (Filzbach 2013) to obtain $P(\theta ,\overset{¯}{y}|y)$.

### 2.4 Sampling effort and error

The continuous time series described in Figure 1 were sampled at discrete times twice a day for the first 4 days, for a total of *d *= 10 observation events, mimicking time series data that may be obtained in realistic field or laboratory experiments (e.g., Acosta, Zamor, Najar, Roe, & Hambright, 2015). Given the growth rate of the introduced species (*r *= 1), the choice of the sampling effort corresponds on average to a survey of the population performed twice per generation time. We set the sampling error to *f *= 0.25 reproducing a search effort of the 25% of the whole habitat.

### 2.5 Precision and accuracy for four simulatedscenarios

The precision and accuracy with which we infer the competition parameter α determine our ability to predict the probability of invasion or extinction of the introduced species. We calculated the precision and accuracy of our estimates of the interaction parameter using the standard error of the inferred distribution for α, and the absolute distance between the mean inferred value of that distribution and the real parameter value used for simulations. Fixing the resident's carrying capacity (*K*_*R*_), process 4 has four free parameters (λ_*I*_, *K*_*I*_, *N*_*I*_, and α; see Equation 5), note that in process 4, $\mu_{I}=\bar{\lambda_{I}}\left(K_{I}-N_{I}\right)/K_{I}$. We tested our ability to estimate α under four different scenarios, where we changed the degree of prior knowledge of single species parameters (see Equation 10) for the introduced species.

1. First, we fixed all the single species parameters (i.e., λ_*I*_, *K*_*I*_, *N*_*I*_) to their real value in likelihood 9. This gives the unrealistic situation of perfect knowledge of the introduced species properties in the residents' habitat, but provides a benchmark with which to compare the effects of uncertainty in our inferred parameters.
2. Second, we put a prior on the carrying capacity, *K*_*I*_, of the introduced species only, corresponding to the scenario in which we know the demographic parameters of the introduced species (i.e., λ_*I*_ and μ_*I*_), but we have less knowledge on how the introduced species would grow in the same environmental conditions of the resident species.
3. Third, we put priors on both *K*_*I*_ and *N*_*I*_, reproducing a perfect knowledge of the growth rate of the introduced species only, and a reduced knowledge of both its carrying capacity and demographic parameters.
4. Fourth, we put priors on all the single species parameters.

We tested the effect of having priors on single species parameters on the precision (Figure 4c,f,i) and accuracy (Figure 4a,b,d,e,g,h) of our estimates of the interaction parameter. We fixed α to have values before bifurcation (Figure 4a–c), at bifurcation (Figure 4d–f) and after the bifurcation (Figure 4g–i) between establishment and extinction in the solution of Equation 1. All priors are log normal distributions centered on the real value of the parameter. Using log normal distributions for all single species parameters, we were able to vary the widths of the priors with the same proportion using a single parameter σ that was allowed to vary in the range 0.01 < σ < 1. We always used a flat uninformative prior for α.

### 2.6 Predictability and predictive ability

**Figure 2:**
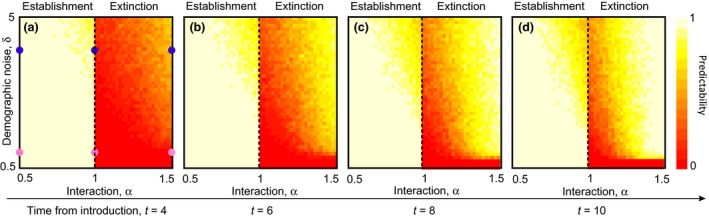
Predictability of establishment as a function of the demographic noise (δ) and of the interaction parameter (α), at different times [*t* = 4 days, panel (a) *t* = 6 days, panel (b) *t* = 8 days, panel (c) *t* = 10 days panel (d)]. The color bar indicates the probability of having the same establishment outcome from the stochastic simulations and the deterministic equations. The probability is computed by simulating 100 replicates of the process 2 starting from with *n*_0_ = 40 individuals. The dashed line indicates the bifurcation point (α = 1) from establishment to extinction in the corresponding deterministic model. The blue and magenta points in panel A represent the corresponding invasion dynamics of Figure 1 for high and low demographic noise, respectively, and for different values of the interaction parameter, α

We quantify the predictability of extinction or establishment at a given time as the probability of observing in the stochastic process the same outcome of the corresponding deterministic solution in the Lotka–Volterra model. We calculated a predictability map of the invasion dynamics for different times, as a function of the demographic noise and of the interaction parameter (0.5 < δ < 5, 0.5 < α < 1.5) (Figure 2).

We quantify our predictive ability as the proportion of times we expect to correctly predict the outcome predicted by long‐term simulation of the deterministic model. This is determined by the proportion of the probability distribution of the interaction parameter, α, that is greater or less than one. For example, if the simulation from which the interaction parameter is inferred has a interaction parameter of 0.5 (i.e., establishment), and all inferred values are less than 1, then all predicted dynamics would correctly predict establishment. If, on the other hand, half of the inferred distribution is greater than 1, then half of predictions are incorrect (those above 1 predict extinction).

**Figure 3:**
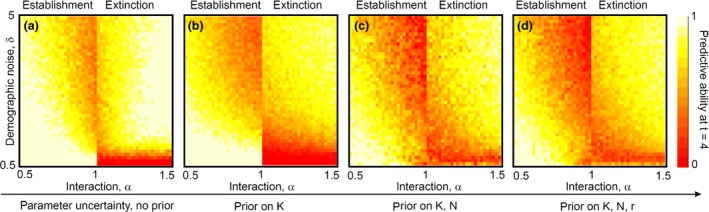
Predictive ability of the inference method of the introduction dynamics as a function of the demographic noise (δ) and the value of the interaction parameter (α). The color‐coded map shows the probability of predicting the deterministic behavior from the Lotka–Volterra equations. The estimates of the inference method are based on 10 equally spaced observations extracted from the stochastic simulations, during the first 4 days (initial condition for the invader *n*_0_ = 40, same as Figure 4). (a) Single species parameters are fixed to the real values used to simulate the data, (b) lognormal prior on *K*, (c) lognormal priors on *K* and *N*, and (d) lognormal priors on *K*,* N* and *r*. Lognormal priors have a width σ = 0.3 (corresponding to the rightmost columns in the boxplots of Figure 4, that is, large uncertainty on the ecological parameters), and a mean centered on the real value. Each pixel shows averages over 30 realizations of the same numerical experiment

The predictive ability of the model is therefore quantified as the proportion of the distribution of the estimated interaction parameter that is contained below/above the bifurcation line at α = 1 for a deterministic invasion/extinction. We computed this proportion/probability testing the four scenarios of prior knowledge of the single species parameters introduced before. Like in the previous cases, first we fixed the single species parameters to their real values (Figure 3a), and then, we put priors on *K*_*I*_ (Figure 3b), on *K*_*I*_ ad *N*_*I*_ (Figure 3c), and on *K*_*I*_, *N*_*I*_ and *r*_*I*_ (Figure 3d). For this set of simulations, we fixed the mean of the prior of the single species parameters to their real value and the width parameter to σ = 0.3.

## 3 Results

### 3.1 Predictability

The predictability of establishment varies greatly depending on demographic parameters (i.e., λ, μ) and the interaction with the resident species (Figure 1). Low demographic stochasticity (red lines) show predictable dynamics, with all populations establishing when the interaction would lead to coexistence in the solution of the deterministic model, or all going extinct when the interaction would lead to extinction in the solution of the deterministic model. Higher demographic stochasticity decreases the predictability of establishment in both cases (blue lines): some populations fail to establish, or fail to go extinct (at least by time = 10), even when the deterministic outcome is establishment or extinction, respectively. Predictability is generally low when the interaction strength puts the dynamics exactly on the bifurcation between establishment and extinction and high demographic stochasticity (δ > 1) flattens the distribution of population densities (Figure 1b,d,f).

Predictability of establishment is further shown in Figure 2, as a function of time, interaction parameter value, and the amount of demographic stochasticity. At time = 4, all introduced populations are still extant, regardless of their ultimate fate; hence, there is very high predictability for all populations that would experience deterministic establishment, and low predictability for those that would experience deterministic extinction (regardless of the strength of demographic stochasticity). By time = 10, there is some reduction in predictability with high demographic noise in the deterministic establishment scenario: This corresponds to some of the populations experiencing stochastic extinction. In the deterministic extinction scenario, predictability is high further from the bifurcation and with higher demographic stochasticity.

### 3.2 Predictive ability

The results so far discussed concern the predictability of the stochastic outcome. We now discuss the impact of that on our predictive ability. Given a single population trajectory, like the cyan curves in Figure 1a,e, the interaction parameter, α, is always inferred imprecisely and influences the predictive ability of the model, that is, how well we can predict the outcome of the introduction.

Predictive ability exhibits the same asymmetry with respect to the interaction parameter: Great demographic stochasticity tends to decrease predictive ability in the deterministic establishment scenario, but increases it in the deterministic extinction scenario (at time = 4) (Figure 3). Predictive ability is lower when the interaction parameter is closer to the bifurcation, and predictive ability is generally lower given greater uncertainty in single species parameters (see Fig. S1 for the difference between the predictability and the predictive ability as a function of the prior knowledge of the single species parameters). We note that predictive ability is always larger than predictability in the extinction case. This is because we estimate predictive ability using our demographic parameter estimates, whereas we measure predictability using stochastic simulations. For the latter, it is often the case that the population has not yet gone extinct even though the mean trajectory is predicted to be heading to extinction.

Our ability to predict that an introduced species will establish from the early stages of an introduction is severely reduced by uncertainties in the demographic parameters, even at low levels of demographic stochasticity. For example, for α = 0.8 and δ = 1.75, our predictive ability at *t* = 4 reduces from 88% for the case in which all the single species parameters are perfectly known (i.e., no prior) to 65% for the case of having priors on all the single species parameter. In order to quantify the performance of our inference framework, we also compared predictability with predictive ability and predictive ability with no priors with predictive ability with priors on three parameters. In Fig. S1, we show the difference between the heat maps in Figures 2a and 3a–d revealing that a model with good predictive ability can perform better than predictability itself (see the negative values on the right sides of the heat maps corresponding to extinction in Fig. S1). In Fig. S2, we show the difference between the heat maps in Figure 3a,d showing the regions of the parameter space where having more information on the demographic parameters affects the predictive ability of the outcome of the introduction. For example, having prior information on the demographic parameters does not change the predictive ability of the model at low demographic noise in the extinction case (see Fig. S2 for low values of δ and for α > 1).

### 3.3 Precision and accuracy

**Figure 4:**
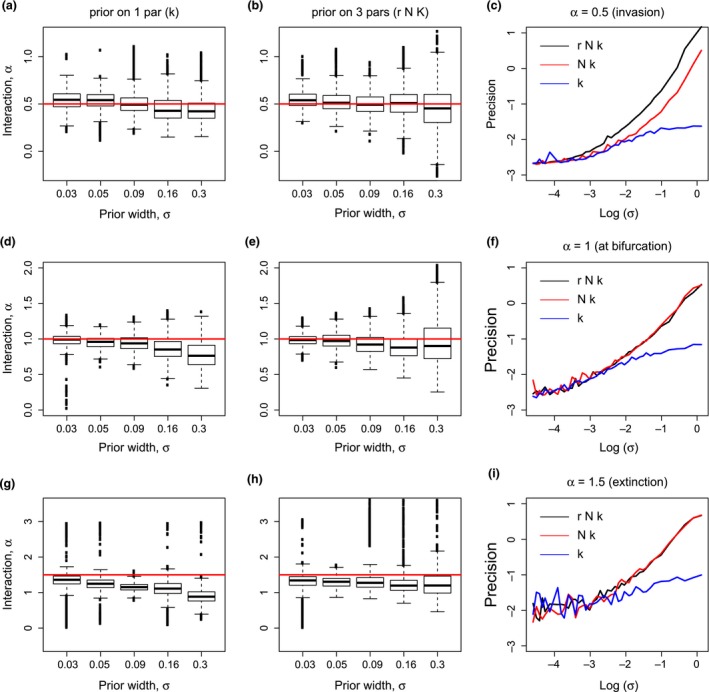
Estimates of the interaction parameter as a function of the information on the ecological traits of the introduced species. The interaction parameter, α, has been fixed to α = 0.5 (a–c), α = 1 (d–f), and α = 1.5 (g–i). Red lines in the first two columns indicate the real value of the interaction parameter. (a, d, g) Box plots of the distributions of the estimates of α were obtained when there is a prior on the single species carrying capacity (*K*) only, and (b, e, h) when there is a prior on all single species parameters (*r*,* N* and *K*). (c, f, i) Log–log plot of the precision of the estimates of α as a function of the parameter σ in the prior, where “precision” refers to the logarithm of the standard error of the estimate of α. Different colors refer to cases where one (*K*; blue), two (*K* and *N*; red), or three (*K*,* N*, and *r*; black) single species parameters are set as priors, with the others fixed to the real value used to simulate the data. The estimates of the inference method are based on 10 equally spaced observations extracted from the stochastic simulations (process 2 during the first 4 days of an invasion, initial condition for the invader *n*_0_ = 40). Plots in panels (c, f, i) show averages over 30 realizations of the same numerical experiment

Limited predictive ability results from demographic stochasticity and imperfect prior knowledge of the single species parameters contributing to uncertainty in the inferred value of the interaction parameter. Imperfect prior knowledge of the single species parameters of the introduced species affects mostly the standard error of the estimates of the interaction parameters, whereas it has little effect on the relative error of the estimate (Figure 4). We observed a consistent underestimation of the interaction parameter as we increase the width of the prior of the single species parameters (Figure 4a–h). The bias in the estimate of the interaction parameter is responsible for the drop below 50% reported in the predictive ability map (Figure 3, panel e). This effect is more pronounced when α > 1 (compare Figure 4a with Figure 4g). This results in a higher predicted probability of detecting an establishment when there is instead extinction, especially at low values of demographic noise (see also the same pattern in the predictability map of Figure 2).

The variance of the estimated interaction parameter as a function of the uncertainty in the carrying capacity of the invader stays below 20% from its real value (i.e., the precision, Figure 4c,f,i, blue lines). On the other hand, when there is also uncertainty in the demographic parameters of the introduced species, the decrease in the precision of the estimate of α, as a function of our the imperfect knowledge of the demographic parameters, is steeper (linear decrease in a log–log plot, compare blue with red and black lines in Figure 4c,f,i). For extremely high accuracy on the parameters (σ < 0.05), the increase in the precision saturates (Figure 4c,f,i). This means that a further decrease in the width of the priors below this value of σ does not substantially contribute to the relative gain in the precision of our estimates of α.

## 4 Discussion

We developed a novel inference method to disentangle the combined effects of demographic stochasticity and parameter uncertainty on our ability to predict the establishment success of an introduced species from the first stages of its introduction. Demographic stochasticity affects not only the probability of establishment (Snyder, 2003; Elliott & Cornell, 2013; Duncan et al., 2014), but also our ability to infer such probability. Our method, based on continuous time stochastic models, enabled us to combine both the effect of ecological factors, such as demographic stochasticity, and the effect of our limited knowledge of the demography of the introduced species (Figures 3 and 4). While the introduction is just the first stage of an invasion and several other factors will affect the introduced species after it establishes (Simberloff, 2011), demographic stochasticity plays a fundamental role in determining the establishment success of the introduced species and therefore affects the whole invasion process. The results from this study provide a quantification of how demographic stochasticity may limit our ability to predict the establishment of introduced species and a quantification of how prior knowledge of the single species parameters can affect our ability to predict the outcome of an introduction.

Our method could be applied to estimate invasion risk in field scenarios, such as lake ecosystems (Drake et al., 2006), where time series data and prior information on the demographic traits of the introduced species are available. As it is not always possible to measure demographic parameters for the introduced species in conditions similar to those of the new habitat, a systematic investigation in controlled invasion dynamics, such as laboratory microcosms experiments (Altermatt et al., 2015; Drake, Huxel, & Hewitt, 1996; Drake & Kramer, 2011), would represent a useful benchmark to test the performance of the model. In fact, our method is suited for predicting establishment success when the single species demographic parameters of the invader are known from previous experiments. In addition, having information on the different stages of replicated invasions of introduced species would enable to directly test the predictions of establishment success that our method provides. Performing experiments to know the demographic parameters of the introduced species in the new habitat would improve the predictive understanding of the invasion process. Experimental tests of our results could help to inform better conservation strategies and eradication programs (Simberloff, 2011). An issue that will arise when applying our framework to field scenarios is that we will need to evaluate whether our model assumptions are likely to be valid. One major assumption we made was that competition only affects birth rates. However, we would expect that this assumption is only valid in some scenarios and in others it might be more realistic to include competitive effects on death rates only or both birth and death rates (Sakai et al., 2001). For example, an invasive bird, in reducing the clutch sizes of resident birds, affects the birth rate of the resident species (O'Connor, Usher, Gibbs, & Brown, 1986). While an invasive plant, out shading competitors will affect the death rate of the residents (Vilà et al., 2011). Adjusting our inference framework to these effects would require a different formulation of the likelihood function based on a more general version of the stochastic Lotka–Volterra birth and death process 2 (see Supplementary Material, section 5.1).

The use of continuous time stochastic models has been proposed for many biological systems, such as evolutionary processes (Kimura, 1964) and epidemics (Alonso, McKane, & Pascual, 2007; Black & McKane, 2010), but their application in ecology is still limited (Ross et al., 2006). Our method, based on a stochastic version of the classic competitive Lotka–Volterra equations, by suitably capturing the inherent transient dynamics of an invasion, would better detect density dependent and environmental effects than inference estimation methods based on deterministic models (e.g., Leslie, 1957; Pascual & Kareiva, 1996). Deriving inference‐based methods using stochastic versions of well‐known deterministic models, such as the Lotka–Volterra competitive equations, has been previously performed (e.g., Kramer & Drake, 2014). Simulating the likelihood function associated with the multispecies stochastic model generally provides a corresponding inference framework (Toni, Welch, Strelkowa, Ipsen, & Stumpf, 2009). With our approach, we used analytical expressions for the mean and variance of population sizes obtained using refined approximations for the stochastic version of the competition model. Given the analytical expressions for the population probability distributions, our method is less computationally expensive than other, recently developed, simulation based, inference methods (e.g., Boys et al., 2007; Toni et al., 2009). The process 4 and the correspondent likelihood function 9 can be further refined in order to include environmental and spatial effects (Drake et al., 2006), and other mechanisms such as the Allee effect (Drake, 2004; Taylor & Hastings, 2005). Furthermore, the approximations developed for the competitive Lotka–Volterra model could also be extended to other types of interactions, such as predator prey interaction (Palamara, Delius, Smith, & Petchey, 2013).

Refined mathematical approximations are useful tools to test and improve inference methods needed to make ecological processes more predictable (Petchey et al., 2015). Given the negative, cumulative impacts at the ecosystem level of invasive species (Cacho et al., 2006; Cardinale et al., 2006; Chapin et al., 2000; Pereira et al., 2010; Simberloff et al., 2013; Vitousek et al., 1996), invasion science raised the challenge toward an improvement of the predictive ability of establishment, and invasion dynamics. Predicting these dynamics is a challenging task, which depend on the understanding of the effects of several, interacting factors. Our inference method, in bridging the gap between species‐centric and community perspective (Lurgi et al., 2014; Simberloff et al., 2013), improves our ability of predicting invasion dynamics (Simberloff, 2011; Simberloff et al., 2013) and represents a general framework that capture the essential ingredients to predict the dynamics of more complex ecological systems.

## Conflict of Interest

None declared.
