# Supplementary figures and images for: The effects of demographic stochasticity and parameter uncertainty on predicting the establishment of introduced species
Source: Ecol Evol. 2016 Oct 27;6(23):8440–51. doi: 10.1002/ece3.2495 (PMC5167034; doi:10.1002/ece3.2495)

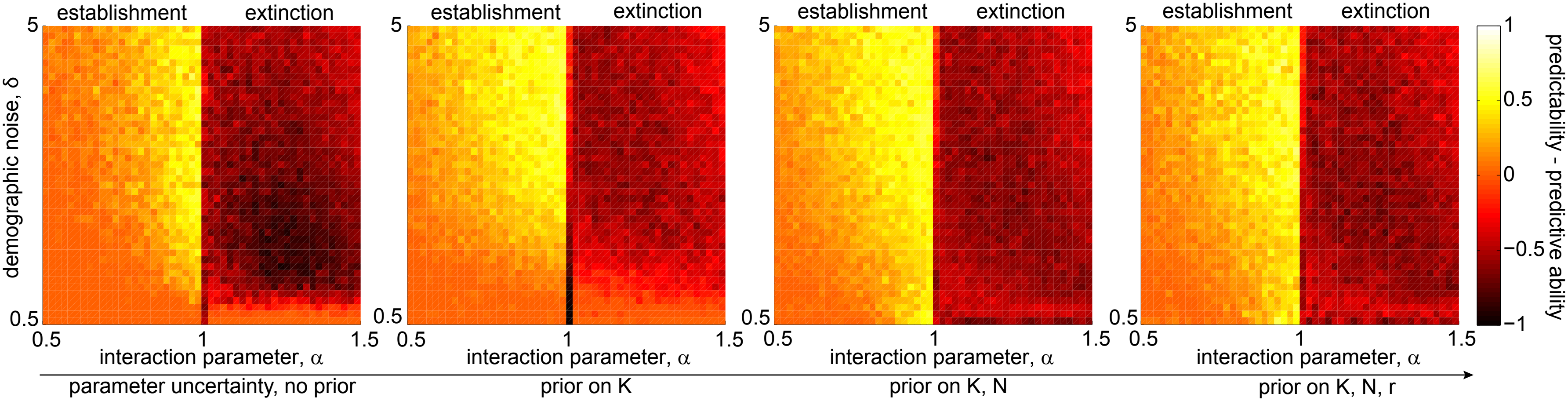

Supplement: Supplementary file 1 [file ECE3-6-8440-s001.pdf]

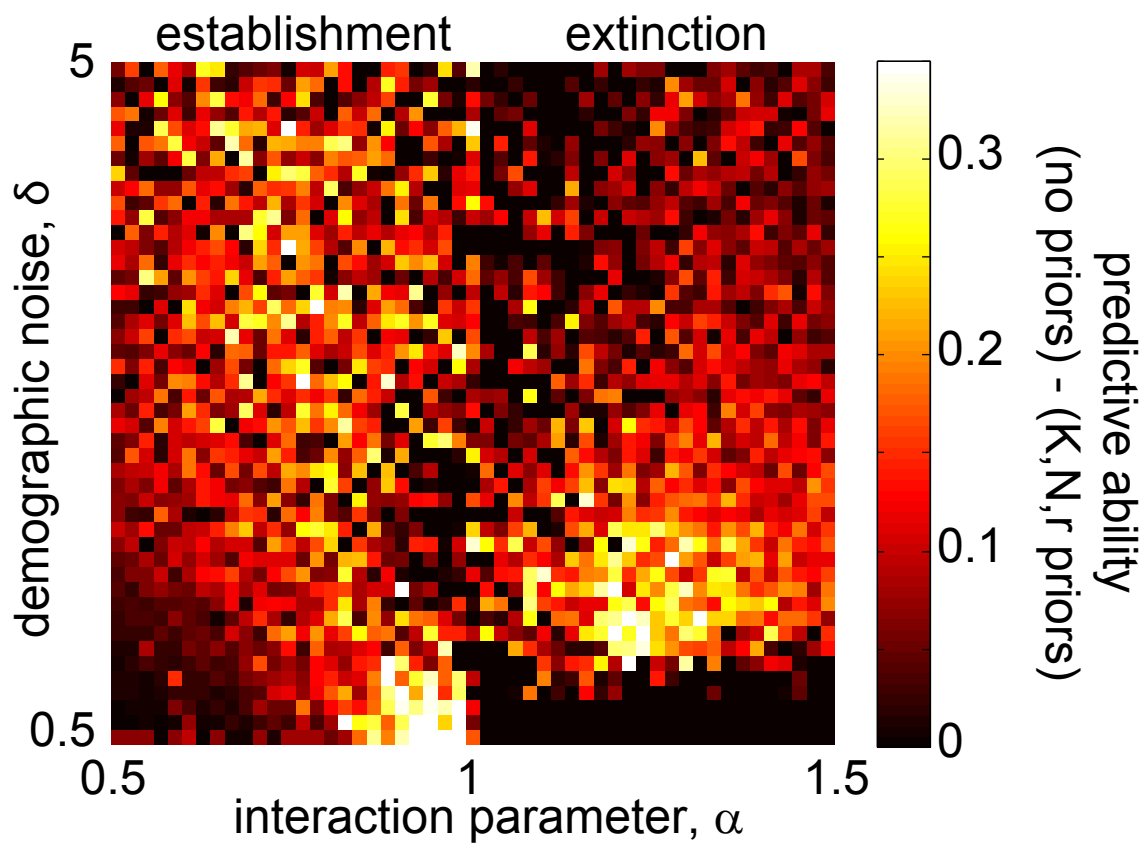

Supplement: Supplementary file 2 [file ECE3-6-8440-s002.pdf]
