# Supplementary material for: The effects of demographic stochasticity and parameter uncertainty on predicting the establishment of introduced species
Source: Ecol Evol. 2016 Oct 27;6(23):8440–51. doi: 10.1002/ece3.2495 (PMC5167034; doi:10.1002/ece3.2495)
Supplement: Supplementary file 3 [file ECE3-6-8440-s003.docx]

# **Supplementary Material**

## **1. General Stochastic Lotka-Volterra BDP**

In this section we introduce a general birth and death processes describing the dynamics of a community of $S$ species competing for the same resources. The linearized process is given by two vectors of population birth and death rates $\boldsymbol{B}(\boldsymbol{n})$ and $\boldsymbol{D}(\boldsymbol{n})$ defined by

$$B_{i}(\boldsymbol{n})=\lambda_{i}n_{i}\left( 1-\frac{\sum_{j}^{S} \alpha_{ij}n_{j}}{N_{i}} \right),$$

|  | $D_{i}(\boldsymbol{n})=\mu_{i}n_{i}\left( 1+\frac{\sum_{j}^{S} \beta_{ij}n_{j}}{N_{i}} \right),$ | (11) |
| --- | --- | --- |

where $0\leq n_{i}\leq N_{i}$ is the (integer) number of individuals of species $i$ ($1\leq i\leq S$), $N_{i}$ is population size at which species $i$ has zero probability of births, $\lambda_{i}$ and $\mu_{i}$ are the birth and death probabilities in the absence of density dependence, respectively (units: $day^{-1}$). Matrices $\boldsymbol{\alpha}$ and $\boldsymbol{\beta}$ contain the per capita effects of competition on births and deaths respectively. The diagonal part of $\boldsymbol{\alpha}$ and $\boldsymbol{\beta}$ contains the intra-specific competition coefficients, while the off-diagonal parts contain the interspecific competition coefficients.

|  | $\frac{dn_{i}}{dt}=r_{i}n_{i}\left( 1-\frac{\sum_{j}^{S} A_{ij}n_{j}}{K_{i}} \right),$ | (12) |
| --- | --- | --- |

where $r_{i}=\lambda_{i}-\mu_{i}$ is the per capita growth rate of species $i$, $K_{i}=r_{i}N_{i}/\lambda_{i}$ is the carrying capacity of species $i$ and $A_{ij}=\alpha_{ij}+\mu_{i}\beta_{ij}/\lambda_{i}$ is the per capita effect of species $j$ on species $i$. The deterministic model 12 can be seen as a first approximation of the stochastic model 11. Setting $\boldsymbol{\beta}=0$ (i.e. there is no effect of competition on the death rate of any species), and in the mean field approximation, we obtain the simpler process 2 used to describe the dynamics of an invasion.

Assuming $n_{j}\equiv K_{j}$ (i.e. species $j$ remains at the fixed averaged density $K_{j}$) process 11 can be linearized into a general Verhulst-like stochastic birth and death process (Nåsell 2001) for species $i$ given by population birth and death rates $B_{i}(n_{i})$ and $D_{i}(n_{i})$ defined by

$$B_{i}(n_{i})=\overline{\lambda}_{i}n_{i}\left( 1-\frac{n_{i}}{N_{i}} \right),$$

|  | $D_{i}(n_{i})=\overline{\mu}_{i}n_{i}\left( 1+\frac{n_{i}}{N_{i}} \right).$ | (13) |
| --- | --- | --- |

The modified intrinsic birth and death rates are given by

$$\overline{\lambda}_{i}=\lambda_{i}\left( 1-\frac{\alpha_{ij}K_{j}}{N_{i}} \right),$$

|  | $\overline{\mu}_{i}=\mu_{i}\left( 1+\frac{\beta_{ij}K_{j}}{N_{i}} \right),$ | (14) |
| --- | --- | --- |

where the only assumption done is that the second order terms ($O(\frac{1}{N_{i}^{2}})$) are negligible. Note that when $\boldsymbol{\beta}=0$ we obtain expression 5.

The associated stochastic differential equation (SDE) of the process 13 is

|  | $\frac{dn_{i}(t)}{dt}=F_{i}(n_{i}(t))+\sqrt{H_{i}(n_{i}(t))}\frac{dW(t)}{dt},$ | (15) |
| --- | --- | --- |

where, from 13, we defined two associated functions $F_{i}(n_{i})=B_{i}(n_{i})-D_{i}(n_{i})$ and $H_{i}(n_{i})=B_{i}(n_{i})+D_{i}(n_{i})$, and where $W$ is the standard Wiener process, where $\Delta W(t)=W(t+\Delta t)-W(t)$ has a normal distribution with 0 mean and variance given by $\Delta t$ (Allen & Allen 2003, Gardinier 2009). The first term in the SDE 15 is the deterministic part, and represent the classic logistic equation (Nåsell 2001). The second term in equation 15 describes the demographic stochasticity.

The discrete counterpart of the continuous process 15 is the following master equation (Gadinier 2009)

|  | $\frac{dP(n_{i},t)}{dt}=D_{i}(n_{i}+1)P(n_{i}+1,t)+B_{i}(n_{i}-1)P(n_{i}-1,t)-H_{i}(n_{i})P(n_{i},t),$ | (16) |
| --- | --- | --- |

where $P(n_{i},t)$ is defined as the probability of having $n_{i}$ individuals of species $i$ at time $t$. In analogy with chemical kinetics, we call the functions $B_{i}$ and $D_{i}$ the reaction hazards and $H_{i}$ the cumulative hazard of the process (Wilkinson 2006). A detailed mathematical analysis of equations like 6 is usually intractable, but is straightforward to simulate the time evolution of the system given the rates 13. We used the Gillespie algorithm as a discrete event simulation procedure (Gillespie 1977).

# **2. Supplementary Figure Captions**

## **2.1. Caption Supplementary Figure 1**

Difference between the predictability at time $t=4$ (figure 2 A) and the predictive ability (figure 3 A, B, C, D) as a function of the demographic noise ($\delta$) and the value of the interaction parameter ($\alpha$). The color coded map shows the difference between the two quantities (range in between -1 and +1). Positive values mean that the predictability of the model is greater than our ability to predict the final outcome of the introduction, because of the uncertainty in the parameters (compare the progressive decrease of predictive ability going from left to right corresponding to increasing our uncertainty in the estimates of the parameters). Negative values correspond to an inference framework with a better predictive ability compared to predictability, meaning that the deterministic outcome of the introduction is predicted from the state of the introduced population at $t=4$.

## **2.2. Caption Supplementary Figure 2**

Difference between the predictive ability at time $t=4$ for the case with no prior (figure 3 A) and the predictive ability with three priors (figure 3 D) as a function of the demographic noise ($\delta$) and the value of the interaction parameter ($\alpha$). The color coded map shows the difference between the two quantities (note the range between 0 and 0.35).

**3. References**

(Allen & Allen 2003) Allen, L. J. S. & Allen, E. J. (2003) A comparison of three different stochastic population models with regard to persistence time. Theoretical Population Biology, 64, 439-449.

(Gardiner 2009) Gardiner, C. (2009) Stochastic methods: a handbook for the natural and social sciences. Springer.

(Gillespie 1977) Gillespie, D. T. (1977). Exact stochastic simulation of coupled chemical reactions. The Journal of Physical Chemistry, 81, 2340-2361.

(Nåsell 2001) Nåsell, I. (2001) Extinction and quasi-stationarity in the Verhulst logistic model. Journal of Theoretical Biology, 211, 11-27.

(Wilkinson 2006) Wilkinson, D. (2006) Stochastic modelling for systems biology. Boca Raton, Chapman & Hall/CRC.
